# Insulin Resistance in Bipolar Disorder: A Real-World Cross-Sectional Study

**DOI:** 10.3390/jpm16010047

**Published:** 2026-01-12

**Authors:** Andrea Aguglia, Matteo Meinero, Valentina Aprile, Tommaso Cerisola, Giuditta Mazzarello, Angelo Oggianu, Alessandra Costanza, Mario Amore, Andrea Amerio, Gianluca Serafini

**Affiliations:** 1 Department of Neuroscience, Rehabilitation, Ophthalmology, Genetics, Maternal and Child Health, Section of Psychiatry, University of Genoa, Largo Paolo Daneo 3, 16132 Genoa, Italy; matteomeinero10@gmail.com (M.M.); valentina.aprile1@gmail.com (V.A.); tomceri8@gmail.com (T.C.); giuditta.mazzarello@hotmail.com (G.M.); mario.amore@unige.it (M.A.); andrea.amerio@unige.it (A.A.); gianluca.serafini@unige.it (G.S.); 2 IRCCS Ospedale Policlinico San Martino, Largo Rosanna Benzi 10, 16132 Genoa, Italy; angelo.oggianu@hsanmartino.it; 3 Department of Psychiatry, Faculty of Medicine, Geneva University (UNIGE), 24 Rue du General-Dufour, 1211 Geneva, Switzerland; alessandra.costanza@unige.ch; 4 Department of Psychiatry, Faculty of Biomedical Sciences, University of Italian Switzerland (USI), via Buffi 13, 6900 Lugano, Switzerland

**Keywords:** bipolar disorder, insulin resistance, metabolic dysfunction, HOMA-IR, clinical severity, precision psychiatry

## Abstract

**Background/Objectives:** Bipolar disorder (BD) is increasingly recognized as a multisystem condition in which metabolic abnormalities, particularly insulin resistance (IR), may be linked to illness severity and neuroprogression. Despite growing evidence linking IR to adverse clinical outcomes, the data is heterogeneous and preliminary, and its specific association in hospitalized patients with BD remains underexplored. **Methods:** This cross-sectional study included 86 inpatients with a primary diagnosis with BD at the IRCCS Ospedale Policlinico San Martino, Genoa, Italy, between July 2023 and January 2024. Sociodemographic, clinical, and metabolic characteristics were systematically investigated. IR was defined as a HOMA-IR index ≥ 2.5. **Results:** Twenty-eight patients met criteria for IR. Insulin resistant patients showed a significantly longer illness duration, more frequent residual symptoms, and higher rates of ≥5 lifetime psychiatric hospitalizations. They also exhibited greater polypharmacy (≥4 psychotropics at discharge) and daily alcohol use. Furthermore, the IR subgroup was significantly associated with higher body mass index and triglycerides, lower HDL cholesterol and physical activity levels. **Conclusions:** Our findings indicate that IR is associated with markers of greater illness burden in BD. While these results are consistent with emerging hypotheses on metabolic dysfunction in BD, longitudinal studies are required to clarify temporal and causal relationships. These associations suggest that IR may represent a clinically relevant component of BD rather than a secondary metabolic consequence. Routine metabolic screening and the preferential use of metabolically neutral agents may improve long-term outcomes and align with the emerging paradigm of precision psychiatry.

## 1. Introduction

Bipolar disorder (BD) is a chronic and recurrent psychiatric illness characterized by alternating (hypo)manic and major depressive episodes, frequently accompanied by residual affective symptoms and functional impairment. BD affects approximately 2% of the general population [1] and typically begins in young adulthood. BD presents marked clinical heterogeneity, encompassing subtypes with more severe courses and poorer treatment response. As highlighted by Oliva et al. [2], this variability underscores the need to identify biological markers that can aid in risk stratification and guides personalized therapeutic approaches. Cardiovascular diseases significantly contribute to reduce the life expectancy by up to 20 years in patients with BD and represent the leading cause of natural death in this population, reflecting a high cardiometabolic comorbidity burden [3,4,5].

### 1.1. Cardiometabolic Comorbidities and Clinical Impact

BD is strongly associated with metabolic abnormalities, including abdominal obesity (51%), type 2 diabetes (9.6%), and metabolic syndrome (MetS) (up to 59%) [6,7]. Dyslipidemia and hypertension affect approximately 40% and 25% of patients, respectively, suggesting an intrinsic cardiometabolic vulnerability [8]. As a matter of fact, these alterations are detectable in young individuals, consisting of an increased rates of MetS between ages 13 and 28 [9] and atherogenic lipid profiles in adolescents without overt obesity [10].

Cardiovascular risk is markedly elevated, related to number of episodes and duration of illness [11], with higher rates of ischemic stroke, ischemic heart disease, heart failure, and all-cause mortality (hazard ratio = 2.18) [12], even after controlling for Charlson Comorbidity Index (CCI), suggesting pathophysiological mechanisms beyond traditional risk factors. Insulin resistance (IR) is increasingly recognized as one such mechanism. IR is a metabolic condition in which normal circulating concentrations of insulin elicit a sub-normal biological response, leading to impaired uptake and utilization of glucose in skeletal muscle, adipose tissue and liver. It is highly prevalent in the general population and, often, precedes over type 2 diabetes, reflecting a systemic metabolic dysfunction. IR is considered a core pathophysiological mechanism, underlying MetS and its components but IR also acts as an independent cardiovascular risk factor, being associated with an increased incidence of major cardiovascular events and all-cause mortality. IR is usually clinically silent and it is commonly quantified in clinical and epidemiological studies using the Homeostasis Model Assessment of Insulin Resistance (HOMA-IR), which is derived from fasting glucose and insulin levels. Using a HOMA-IR threshold > 2.35, IR is found in 40.8% of patients with BD vs. 24.7% of healthy controls [13]. This has been observed even in drug-naïve individuals, suggesting IR may represent an early metabolic marker of BD. IR, a key driver of MetS, independently increases cardiovascular risk (hazard ratio = 1.67) and may precede the onset of metabolic comorbidity [14,15]. Metabolic abnormalities also appear linked to clinical severity. Dyslipidemia shows state-dependent variations, with lipid profiles fluctuating across manic and mixed phases [10,16]. Patients with obesity, MetS, or type 2 diabetes experience more frequent affective episodes, greater mood instability, lower remission rates, and poorer functioning [7,17,18], suggesting a clinical subgroup with both metabolic alterations and more severe disease trajectories.

### 1.2. External Factors

Pharmacological treatment significantly contributes to metabolic deterioration. Among mood stabilizers, lithium and lamotrigine are generally metabolically neutral, although lithium doubles the risk of hypothyroidism and increases chronic kidney disease risk [19]. Valproate is associated with weight gain in up to 50% of patients, particularly in women [20]. Clozapine and olanzapine have the most pronounced metabolic impact, leading to weight gain, hyperglycemia, and hypertriglyceridemia within six weeks of treatment [21]. Notably, olanzapine worsens HOMA-IR even without weight gain, suggesting a direct effect on insulin sensitivity [22]. Antidepressants have a milder metabolic profile, though mirtazapine induces early weight gain, whereas bupropion promotes weight and waist circumference reduction [23]. Lifestyle and psychiatric comorbidities further exacerbate metabolic risk. Patients with BD exhibit unhealthy lifestyles such as lower physical activity, poorer diet quality, sleep disturbances, and higher smoking rates, all factors that can affect negatively the metabolic abnormalities [24].

### 1.3. Pathophysiological Hypotheses

IR may be associated with bipolar pathophysiology by influencing central and peripheral systems. Low-grade chronic inflammation, consistently observed even in bipolar youth [25,26], is linked to higher interleukin (IL)-6, c-reactive protein (CRP), and tumor necrosis factor (TNF)-α levels, correlating with more severe affective symptomatology [27]. IR may amplify this inflammatory response through persistent cytokine secretion from adipose tissue and intracellular signaling dysfunction [28,29]. Hypothalamic–pituitary–adrenal (HPA) axis dysregulation can contribute as a shared mechanism, with altered cortisol levels related to a greater clinical severity [30,31,32]. IR may contribute to HPA hyperactivation by promoting cortisol overproduction and endocrine imbalance [33,34]. Mitochondrial dysfunction, a hallmark of BD, involves impaired oxidative phosphorylation, increased oxidative stress, and disrupted biogenesis, even in euthymia [35]. IR may play a role in all these alterations [36,37]. Dopaminergic dysregulation has also been implicated, with higher body mass index (BMI) associated with reduced dopaminergic gene expression [38] and IR is linked to increased dopamine turnover and reduced mesolimbic signaling efficiency [39,40].

Reduced peripheral brain-derived neurotrophic factor (BDNF) levels are reported in BD [26,41]. Chronic hyperglycemia, typical of IR, suppresses cerebral BDNF production [42], leading to impaired synaptic plasticity and depressive-like behaviors resistant to treatment [43,44].

Together, chronic inflammation, HPA hyperactivation, oxidative stress, mitochondrial dysfunction, dopaminergic dysregulation, and reduced neurotrophic signaling could drive neuroprogression in BD [45,46,47]. The involvement of IR in each of these pathways supports its potential role as a factor associated with disease neuroprogression. Blood–brain barrier dysfunction-linked to more severe clinical profiles, higher BMI, elevated HOMA-IR, hypertriglyceridemia, and hypertension may represent a neuroprogressive marker influenced by IR through endothelial injury and disrupted insulin signaling [48,49]. However, findings relating IR to illness neuroprogression in BD are preliminary and require cautious interpretations.

### 1.4. Study Rationale

Overall, IR emerges as a potential cross-sectional modulator of multiple pathophysiological mechanisms underlying bipolar severity and neuroprogression. Its clinical characterization could be essential to clarify its impact on illness trajectory and assess its value as a marker of clinical severity. Given its strong association with cardiovascular risk, systematic evaluation of IR in BD could represent a relevant preventive step. Accordingly, the aim of this study was to investigate the association between IR and indicators of clinical severity in BD, through a systematic assessment of sociodemographic, clinical, and metabolic characteristics in a cohort of inpatients with a primary diagnosis of BD.

## 2. Materials and Methods

### 2.1. Sample

This study was conducted on inpatients with a primary diagnosis of BD, consecutively admitted to the Psychiatry Unit of the IRCCS Ospedale Policlinico San Martino, Department of Neuroscience, Rehabilitation, Ophthalmology, Genetics, and Maternal and Child Health, University of Genoa, Italy, between July 2023 and January 2024. The diagnosis of BD was established according to the criteria of the Diagnostic and Statistical Manual of Mental Disorders, Fifth Edition—text revised [50]. 

The inclusion criteria were (a) admission to our psychiatric ward; (b) current age ≥ 18 years; (c) willingness to participate in the study. The exclusion criteria were (a) the presence of schizophrenia and related disorders; (b) the presence of current pregnancy or recent childbirth; (c) history of neurological disorders such as neurodegenerative diseases, loss of consciousness due to neurological causes, or intellectual disability; (d) use of hypoglycemic agents or diagnosis of type 2 diabetes; (e) refusal or inability to provide valid informed consent.

All recruited patients voluntarily agreed to participate in the study, provided a written informed consent, received detailed information about the study objectives and procedures, and was given the opportunity to ask questions. The study protocol was conducted in accordance with the principles of the latest version of the Declaration of Helsinki [51] and was approved by the Institutional Review Board of IRCCS Ospedale Policlinico San Martino (Genoa).

### 2.2. Assessments and Procedure

Participants were considered euthymic at the moment of evaluation, as indicated by subthreshold Hamilton Depression Rating Scale (<8) and Young Mania Rating Scale (<6).

Sociodemographic and clinical characteristics of the total sample were analyzed and documented during hospitalization, using both paper-based and electronic medical records routinely employed in our psychiatric ward. These records included detailed medical history and standard clinical information through a semi-structured interview used in previous studies [52,53,54]. Sociodemographic data included age, sex, marital status, occupational status, educational level, living condition and having children. The clinical characteristics included BD (type I and II, cyclothymia), age at onset, bipolar cycle, predominant polarity, illness duration, presence of psychiatric and medical comorbidity, number and characteristics of previous hospitalizations, and current pharmacological treatments. Polypharmacotherapy (≥4 psychotropic medications) was defined according to the clinical definition of ‘complex polypharmacy’ used in psychiatric research. Exposure to antipsychotic medication was recorded as a binary variable (yes/no), as the distribution across individual agents and dosages did not allow for meaningful stratification by specific antipsychotic subclass. Bipolar cycle was classified as depressive–mania–interval (DMI) or mania–depression–interval (MDI), according to established criteria [55]. Patients whose pattern did not meet either definition were categorized as ‘irregular’, reflecting heterogeneous longitudinal trajectories. Furthermore, participants were also asked about their level of physical inactivity or engagement in regular physical activity. Regular physical activity was defined as absent, mild (4 h/week), moderate (around 4 h/week), and intense (>4 h/week) as captured by the semi-structured clinical interview routinely used in our unit.

Alcohol consumption was also assessed and categorized as ‘daily’ versus ‘non-daily’; detailed patterns such as quantity or binge drinking were not systematically recorded but daily use was confirmed by at least four alcohol units. Information on smoking status and general dietary habits was gathered qualitatively during clinical assessment.

Psychiatric comorbidities were systematically assessed and included alcohol use disorder, other substance use disorders (primarily cannabis and cocaine), and anxiety disorders. Medical comorbidities beyond type 2 diabetes were documented, including hypertension, dyslipidemia, and thyroid dysfunction, due to their potential impact on metabolic status and hospitalization patterns. An acute medical condition was considered as an exclusion criterion.

Upon admission to the psychiatric ward, all participants underwent routine blood sampling. Blood samples were collected between 7:00 and 8:30 a.m. after an overnight fast of at least 10 h and following an initial psychiatric evaluation. For patients who were not fasting, the procedure was postponed. In addition to standard laboratory parameters, fasting insulin, glucose, total cholesterol (TC), triglycerides (TG), high-density lipoprotein cholesterol (HDL-c), and low-density lipoprotein cholesterol (LDL-c) were measured. Samples collected during hospitalization were analyzed at the laboratory of the IRCCS Ospedale Policlinico San Martino, Genoa, Italy.

To evaluate the presence and degree of IR, the Homeostasis Model Assessment (HOMA) index was calculated using the following formula:

$$HOMA-IR=\frac{Fasting\;insulin\;\left(\frac{\mu U}{mL}\right)\;*\;Fasting\;glucose\;\left(\frac{mg}{dL}\right)}{405}$$

The choice of this parameter was based on its wide validation in the literature and its established use in both clinical practice and research. Originally introduced by Watt et al. [56] and later confirmed as a reliable measure of insulin sensitivity [57], the HOMA model provides a robust and non-invasive estimate of IR. In line with previous studies, a HOMA-IR value ≥ 2.5 was considered indicative of IR. The HOMA-IR correlates well with estimates using the euglycemic clamp method and it is a well-accepted measure of IR [58]. This threshold has been widely adopted in European clinical cohorts and psychiatric populations, including bipolar samples, where HOMA-IR cut-offs between 2.0 and 2.5 are commonly used [59,60,61]. Accordingly, the cut-off of 2.5 was selected as a clinically and methodologically consistent value for defining IR in our sample. Anthropometric measures included BMI, calculated as weight in kilograms divided by height in meters squared (kg/m^2^); this parameter was used to identify overweight (25 ≤ BMI ≤ 29.9) and obese (BMI ≥ 30) individuals.

Glycemic status beyond fasting glucose was not formally assessed, as participants were classified solely based on HOMA-IR values; no oral glucose tolerance testing was performed while HbA1c measurements were normal in all patients.

### 2.3. Statistical Analysis

Statistical analysis was performed using the Statistical Package for the Social Sciences (SPSS), version 25.0 (IBM Corp., Armonk, NY, USA). A statistical significance was set at *p* value < 0.05.

Continuous variables were expressed as mean ± standard deviation (SD), while categorical variables were presented as frequencies and percentages. The Kolmogorov–Smirnov test was used to assess the normality of variable distributions. The total sample was divided in two subgroups according to the presence of IR. Comparisons with continuous variables were performed using the independent-samples *t*-test, whereas categorical variables were compared using the chi-square (χ^2^) test. Finally, we used a stepwise regression analysis with backward selection, i.e., we started by including all candidate variables and tested how the deletion of a variable affected the statistical significance of the fit. The following variables were selected a priori as candidate predictors based on clinical relevance and statistical significance at univariate analysis: irregular bipolar cycle, presence of residual symptoms, number of hospitalizations > 5, daily alcohol use, presence of polypharmacotherapy, weight in kg, body mass index, physical activity, HDL cholesterol, triglycerides, corrected for current age and sex.

Given the limited number of cases with IR (N = 28), the regression analysis was intended as an exploratory and hypothesis-generating approach rather than a fully powered multivariable model. This approach was intended to eliminate potential collinearities among different predictors of IR. All significant variables were reported by providing their odds ratio (OR) and confidence interval (CI) 95%.

HOMA-IR was also examined as a continuous variable, and the pattern of results remained consistent with those obtained using the dichotomous threshold. Age and living condition were evaluated as potential confounders but were not included in the regression model because they did not differ significantly between groups and did not demonstrate meaningful associations with metabolic or clinical outcomes.

This study was conducted in accordance with the STROCSS (Strengthening the Reporting of Cohort, Cross-sectional and Case–control Studies) guideline from the EQUATOR Network.

## 3. Results

### 3.1. Sociodemographic Variables

A total of 86 patients with BD were enrolled in this study, of whom 28 exhibited a HOMA-IR ≥ 2.5 and were classified as IR. The mean current age of the sample was 49.17 ± 15.03 years. All sociodemographic characteristics are reported in Table 1.

### 3.2. Clinical and Metabolic Variables

Regarding the diagnosis, 39 patients were BD type I (45.3%), 36 were BD type II (41.9%), and 11 were cyclothymia (12.8%).

A different distribution of bipolar cycle was observed between the two subgroups. Among bipolar patients with IR, irregular bipolar cycle was more frequent (75% vs. 48%) and, when a bipolar cycle was defined, mania-depression-interval (MDI) cycle was more observed in patients without IR. In bipolar patients with IR, the duration of illness was significantly longer compared to patients without IR (26.13 ± 14.09 years vs. 18.72 ± 12.59 years, *p* = 0.028) as well as a higher prevalence of residual symptoms (53.6% vs. 31%, *p* = 0.044) and a number of hospitalizations > 5 (57.1% vs. 25.9%, *p* = 0.005). Finally, the proportion of patients both receiving polypharmacotherapy (≥4 medications) and reporting daily alcohol use were more prevalent in patients with IR. A history of psychotic symptoms was present in 29 patients (33.7%), with similar proportions in the two subgroups (39.3% vs. 31.0%).

Regarding metabolic parameters, bipolar patients with IR led a more sedentary lifestyle, with a lower prevalence of physical activity (28.6% vs. 56.9%, *p* = 0.014) and a higher body weight (80.00 ± 16.73 vs. 67.99 ± 12.38, *p* < 0.001). Furthermore, the insulin-resistant subgroup had lower HDL-c (40.9 ± 16.19 mg/dL vs. 54.56 ± 17.20 mg/dL, *p* = 0.003) and higher TG levels (160.82 ± 82.37 mg/dL vs. 105.58 ± 53.56 mg/dL, *p* = 0.009) compared to the patients without IR. In the total sample, mean HOMA-IR was 2.34 ± 2.27, with a median of 1.58 and interquartile range (IQR) 1.01 (25%)–2.92 (75%). As expected, patients with IR displayed significantly higher HOMA-IR values than non-IR patients. All clinical and metabolic characteristics are summarized in Table 2.

A stepwise logistic regression analysis with IR in patients with a primary diagnosis of BD as the independent variable generated the following ORs: 6.405 for number of hospitalizations > 5 (CI 1.238–33.148; *p* = 0.027) and 1.450 for BMI (CI 1.067–1.970; *p* = 0.018) with R^2^ Nagelkerke = 0.578. Detailed model outputs are available on Supplementary Materials (Table S1).

## 4. Discussion

### 4.1. Main Findings

In this study, the association between IR and clinical-metabolic characteristics in patients with BD was investigated. Our findings indicate that IR is significantly associated with markers of greater illness severity, including a higher number of psychiatric hospitalizations, longer illness duration, more frequent residual symptoms, and increased polypharmacotherapy at discharge, related to a greater difficulty in mood stabilizing. Several alternative explanations should be considered as potential risk factor, exacerbating metabolic dysfunction and greater illness severity such as cumulative exposure to medications affecting weight and insulin sensitivity, irregular lifestyle habits, chronic stress, and sleep disruption. Conversely, early-life or genetic vulnerabilities predisposed to IR might affect neurodevelopment and, thereby, contribute to more severe bipolar trajectories. Given the cross-sectional design, the directionality of these associations cannot be determined.

Epidemiological studies indicate a bidirectional association between BD and IR. IR is observed in approximately 24–41% of individuals with BD, including drug-naïve or early-stage patients, while individuals with metabolic disorders exhibit higher rates of BD. This reciprocal pattern reinforces the hypothesis that IR may represent a clinically relevant dimension within bipolar illness rather than a secondary metabolic consequence, resulting from pharmacological treatment or unhealthy lifestyles [13,62,63].

Recent research highlights the overlap between metabolic and affective dysregulation, emphasizing chronic low-grade inflammation, oxidative stress, mitochondrial dysfunction, and HPA axis hyperactivity as shared biological mechanisms [30,35,64,65]. This convergence reinforces the hypothesis that BD and metabolic disorders share a systemic vulnerability, where IR acts both as a biomarker and a clinical correlate potentially linked to bipolar neuroprogression.

### 4.2. Clinical Correlates

The association between IR and daily alcohol consumption observed in univariate analyses should be interpreted cautiously. Daily/non-daily categorization does not capture important distinctions such as binge patterns, quantity, or chronicity of use. Alcohol use disorder is also highly prevalent in BD and may confound simple frequency-based measures. Moreover, this association did not persist in the multivariable analysis, where BMI and illness chronicity accounted for most of the variance. Therefore, the link between IR and alcohol use in this study should be interpreted in a cautious manner. The comorbidity between BD and alcohol use disorder is well established, with lifetime prevalence rates reaching 40–70% [66]. This relationship appears bidirectional: early alcohol exposure may increase vulnerability to BD, while mood instability may foster maladaptive alcohol consumption [67]. Alcohol-induced alterations in glucose metabolism, inflammatory cytokine activity, and hepatic insulin signaling could further exacerbate systemic IR [68].

In our cohort, IR was also linked with a greater number of hospitalizations and persistent residual symptoms; however, in the multivariable model, only BMI and lifetime hospitalizations > 5 remained significantly associated with IR, suggesting that other univariate associations may be partly explained by obesity and illness chronicity. These variables are recognized indicators of clinical complexity and poor long-term outcomes [47,69]. The persistence of subsyndromal depressive symptoms during euthymia is associated with reduced psychosocial functioning, impaired cognition, and higher relapse rates [70]. The longer illness duration observed among patients with IR supports previous findings of a progressive metabolic-psychiatric interplay throughout the course of BD [21,71], although the directionality of this association cannot be inferred from cross-sectional data.

### 4.3. Pharmacological and Metabolic Considerations

Polypharmacotherapy was more frequent among patients with IR, reflecting greater therapeutic complexity. This observation aligned with prior research linking polypharmacotherapy to metabolic disturbances and treatment resistance [24,69]. SGAs, particularly olanzapine and clozapine but also quetiapine, exert well-documented effects on weight gain, dyslipidemia, and IR through histaminergic (H_1_) and serotoninergic (5-HT_2C_) receptor antagonism, as well as mitochondrial and inflammatory pathways [20,72,73]. The sample size did not allow stratification by specific antipsychotic class or dose, nor a sensitivity analysis excluding high-risk antipsychotics. This limitation prevents from fully disentangling treatment effects from intrinsic illness severity.

Recent works by Pillinger et al. [21] and Chow et al. [71] confirmed that SGAs differed markedly in metabolic risk, with clozapine and olanzapine conferring the highest risk, while lurasidone, cariprazine, and aripiprazole remained metabolically neutral [74]. Olanzapine showed to impair insulin signaling via GLUT4 inhibition and mTORC1 activation, leading to intracellular lipid accumulation and systemic IR [75]. In line with previous translational studies [45,76], pharmacological complexity itself may contribute to IR through cumulative mitochondrial and endocrine stress. Therefore, personalized pharmacological strategies should prioritize metabolically neutral agents and include routine metabolic monitoring, particularly in patients with pre-existing IR or other cardiometabolic risk factors.

In this context, several clinical considerations for psychotropic medication selection could be put forward: although our study was not designed to directly compare medication effects, existing evidence indicates that psychotropic agents differ substantially in their metabolic liability. Mood stabilizers such as lithium and lamotrigine, and several SGAs with a more favorable metabolic profile (e.g., lurasidone, cariprazine, aripiprazole), are generally considered to have lower impact on weight, lipid metabolism, and insulin sensitivity. In contrast, antipsychotics with higher metabolic burden, including olanzapine and clozapine, have been consistently associated with significant weight gain, dyslipidemia, and IR. Valproate, although effective as a mood stabilizer, has also been linked to dose-dependent weight gain and may warrant caution in metabolically vulnerable patients. These considerations underscore the importance of integrating metabolic risk assessment into psychopharmacological decision-making, especially in individuals with pre-existing IR or elevated cardiometabolic risk. While such clinical reasoning aligns with precision psychiatry principles, controlled comparative studies are still needed to establish formal treatment recommendations.

### 4.4. Lifestyle and Behavioral Factors

Consistent with prior literature, bipolar patients with IR exhibited higher BMI, lower HDL-c, and higher TG, core components of MetS [33]. They also reported lower levels of physical activity, corroborating findings from Firth et al. [24], who demonstrated that reduced exercise and poor diet quality contributed to both affective and metabolic dysregulation in BD. Unfortunately, physical activity was assessed through self-report, which may be influenced by recall and social desirability bias, particularly in an inpatient population. Dietary habits, smoking status and sleep quality—important determinants of metabolic health—were not systematically recorded and may have contributed to residual confounding. Future studies will incorporate validated lifestyle questionnaires and, when feasible, objective measures such as accelerometry. Lifestyle factors such as sedentary behavior, high-calorie diets, smoking, and poor sleep quality are highly prevalent in BD and act synergistically with neurobiological mechanisms, such as inflammation and dopaminergic dysregulation, to exacerbate illness burden and severity [34,77].

**Figure 1:**
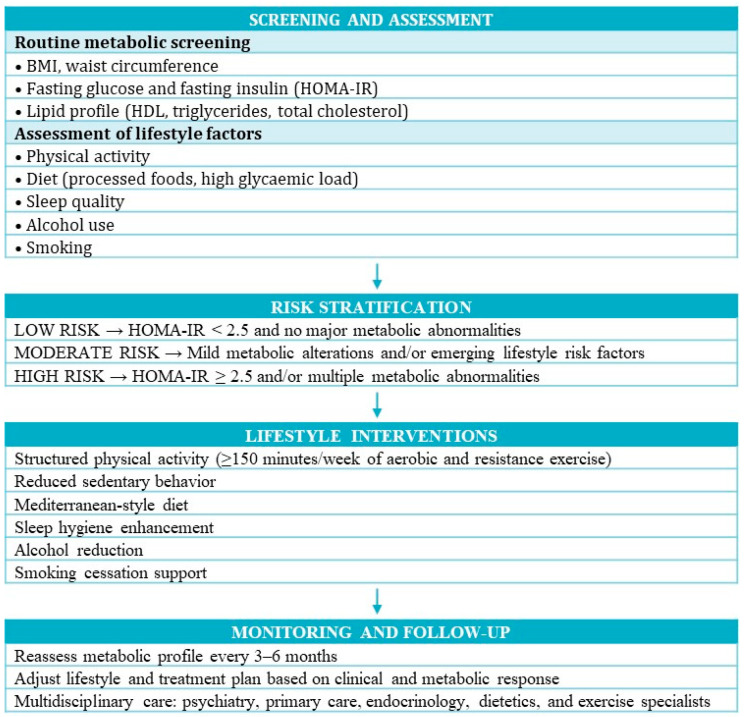
Proposed clinical management pathway for patients with BD at risk for IR.

BD is also characterized by marked circadian rhythm disruption, with alterations in sleep–wake patterns, social rhythms and chronotype. These disturbances may contribute to IR by inducing misalignment between behavioral cycles (e.g., feeding, activity) and endogenous circadian clocks, regulating glucose and lipid metabolism. Experimental and clinical research showed that circadian misalignment could impair insulin sensitivity and promote central adiposity. Recent models conceptualized BD as a “whole-body” disorder in which metabolic and circadian dysregulation are tightly intertwined [78,79]. Sleep disturbances represent a further pathway linking BD and metabolic dysregulation. Short or fragmented sleep, insomnia and hypersomnia—frequently observed across mood states—have been shown to alter HPA axis activity and appetite-regulating hormones such as cortisol, leptin and ghrelin, thereby increasing appetite, promoting weight gain and decreasing insulin sensitivity [80,81]. In our sample, IR was associated with lower levels of regular physical activity, which is consistent with this broader picture of lifestyle-related metabolic risk in BD. However, these factors remain important unmeasured contributors (e.g., diet composition, meal timing, sleep parameters) in our study but could partly account for the associations observed between IR and illness severity (Figure 1).

This multidimensional interaction supports the concept of BD as a systemic disorder characterized by reciprocal reinforcement between metabolic and affective pathways.

### 4.5. Pathophysiological Implications

Recent evidence supports a potential role of IR in brain dysfunction. Insulin signaling regulates synaptic plasticity, neurogenesis, and cerebral energy metabolism; its impairment reduces neuronal glucose uptake, increases oxidative stress, and alters neuroinflammatory tone. These processes may contribute to reduced neuroplasticity and affective dysregulation, as observed in BD. Furthermore, chronic IR has been associated with HPA axis hyperactivation, elevated cortisol levels, and impaired glucocorticoid feedback regulation [7,18]. These alterations could heighten stress sensitivity and mood reactivity, promoting a vicious cycle between metabolic and affective dysregulation. The combination of neuroendocrine, inflammatory, and metabolic disturbances represents a biologically plausible framework that may explain the observed associations between IR and clinical severity in BD. However, these mechanisms derive largely from preclinical and translational research and cannot be inferred causally from the present cross-sectional findings. Alternative explanations—such as systemic inflammation, oxidative stress, endocrine dysregulation, or genetic predispositions—may contribute to both IR and illness severity, underscoring the need for longitudinal and interventional studies to clarify temporal and mechanistic relationships.

Several limitations should be acknowledged. First, cross-sectional design prevents any inference about causality between IR and the clinical severity of BD. Longitudinal and interventional studies will be required to determine whether IR precedes, follows, or co-develops with bipolar neuroprogression and pharmacological exposure. Second, the sample size, single-center recruitment and inpatient setting may limit the generalizability of our findings. Third, we did not include standardized assessments of dietary intake, meal timing or sleep–wake patterns. Although we collected several information on physical activity, smoking and alcohol consumption, the lack of detailed lifestyle measures means that residual confounding by diet, circadian disruption and sleep disturbances cannot be excluded. These factors are increasingly recognized as key determinants of both BD course and IR; therefore, future longitudinal studies should incorporate validated nutritional and sleep instruments to clarify their specific contribution. Fourth, IR was assessed using the HOMA-IR index, applying the commonly used cut-off of HOMA-IR ≥ 2.5, an indirect yet validated measure that can be affected by factors such as stress level or fasting duration. However, it should be acknowledged that optimal HOMA-IR thresholds are not universal and may vary across populations according to age, sex, and ethnicity; therefore, prevalence estimates and associations should be interpreted considering this potential cut-off heterogeneity [82,83]. To mitigate this potential source of measurement bias, blood samples were collected early in the morning after a ≥10 h fast, and all participants were evaluated outside of acute mood episodes, which reduces the likelihood of stress-related fluctuations in glucose and insulin levels, although hospitalization itself may still influence metabolic parameters through acute stress, temporary changes in diet, reduced physical activity, or medication adjustments. Future studies should complement HOMA-IR with additional glycemic indices to broaden metabolic profiling and improve insulin sensitivity assessment. Fifth, although the effects of pharmacotherapy on metabolism were statistically controlled, residual confounding cannot be entirely excluded. Detailed stratification by antipsychotic class and dosage was not conducted, and exposure to high-risk SGAs may contribute to residual metabolic confounding. Future extensions of this project will incorporate more granular pharmacological data and allow for stratified and sensitivity analyses. Sixth, lifestyle variables were self-reported, which may have introduced recall bias and potential underreporting of unhealthy behaviors. Lifestyle factors, including diet, sleep quality, and smoking, were not assessed with standardized tools and may have influenced both metabolic and clinical outcomes, introducing potential residual confounding. Associations such as that between IR and daily alcohol use should be considered exploratory, as the simplified frequency measure used does not capture alcohol severity or patterns (e.g., binge drinking). Larger samples with more granular lifestyle assessments are needed to clarify these relationships. Finally, unmeasured biological factors, including inflammatory markers, cortisol levels, oxidative stress indices, and genetic predispositions, may also have influenced the observed associations. Despite these limitations, the present study provides empirical and meaningful evidence supporting the clinical relevance of IR in BD and underscores the importance of future longitudinal, multimodal research integrating metabolic (e.g., oral glucose tolerance), inflammatory (e.g., interleukin, cortisol levels, oxidative stress), and neuroimaging biomarkers to clarify temporal and mechanistic relationships. Longitudinal and interventional studies—including lifestyle-based interventions and trials of insulin-sensitizing agents—will be essential to determine whether modifying IR results in improvements in the bipolar illness course and to establish temporal precedence and treatment responsiveness.

## 5. Conclusions and Clinical Implications

This study provides empirical evidence that IR is not merely a metabolic comorbidity, but it is associated with clinically significant manifestations of BD. Its strong association with key indicators of illness severity, such as multiple hospitalizations, longer illness duration, residual symptoms, and complex pharmacotherapy suggests that metabolic dysfunction may be linked to the heterogeneity and chronicity of the disorder.

Early identification and management of IR could represent a feasible and cost-effective strategy to improve both psychiatric and physical outcomes. A pragmatic diagnostic pathway for IR in BD should include: (i) systematic measurement of weight, BMI, waist circumference and blood pressure at baseline and regular follow-up visits; (ii) baseline and periodic laboratory tests, including fasting plasma glucose, fasting insulin and a standard lipid profile; and (iii) calculation of the HOMA-IR index when fasting glucose and insulin are available, using established cut-offs of IR. Patients with IR or with clustered metabolic abnormalities (e.g., increased waist circumference, dyslipidemia, elevated blood pressure) should be referred to primary care physicians or endocrinologists for comprehensive cardiovascular and metabolic risk evaluation and management. In patients who already exhibit IR or multiple metabolic risk factors, clinicians may consider, when clinically feasible, the preferential use of psychotropic agents with a more favorable metabolic profile. Furthermore, evidence-based recommendations include structured aerobic and resistance physical activity at least 150 min per week, reduction in sedentary time, and gradual incorporation of routine daily movement. Nutritional counselling focuses on Mediterranean-style dietary patterns, reduced intake of ultra-processed foods and high-glycemic-load carbohydrates, and optimization of meal regularity can further support metabolic health. Addressing modifiable behaviors—such as smoking cessation, moderation of alcohol use, and improvement of sleep hygiene—also represents an important component of care [84]. Importantly, lifestyle interventions are most effective when delivered through collaborative, multidisciplinary care involving psychiatrists, primary care clinicians, endocrinologists, dietitians, and exercise specialists. Therefore, integrating metabolic monitoring and lifestyle guidance into the standard treatment pathway may support personalized care and improve long-term outcomes in individuals with BD.

## Figures and Tables

**Table 1:** Sociodemographic characteristics of sample and according to the presence of IR.

N (%) or Mean ± SD	Total Sample (N = 86)	BD with IR (N = 28)	BD Without IR (N = 58)	X^2^/t	*p*
Gender (male)	37 (43.0)	13 (46.4)	24 (41.4)	0.196	0.658
Current age (years)	49.17 ± 15.03	53.14 ± 15.65	47.26 ± 14.46	−1.721	0.089
Marital Status Single Married Separated/Divorced Widowed	35 (40.7) 28 (32.6) 19 (22.1) 4 (4.6)	13 (46.4) 8 (28.6) 6 (21.4) 1 (3.6)	22 (37.9) 20 (34.5) 13 (22.4) 3 (5.2)	0.650	0.885
Educational level (years)	12.24 ± 3.68	12.21 ± 3.55	12.26 ± 3.76	0.052	0.959
Occupational status, employed	33 (38.4)	9 (32.1)	24 (41.4)	0.681	0.409
Living condition Alone Family Therapeutic Community	26 (30.2) 55 (64.0) 5 (5.8)	9 (32.1) 15 (53.6) 4 (14.3)	17 (29.3) 40 (69.0) 1 (1.7)	5.875	0.053
Having child	51 (59.3)	17 (60.7)	34 (58.6)	0.034	0.853

**Table 2:** Clinical and metabolic characteristics of sample and according to the presence of IR.

	Total Sample (N = 86)	BD with IR (N = 28)	BD Without IR (N = 58)	X^2^/t	*p*
Clinical Characteristics, N (%) or Mean ± SD					
Psychiatric family history	54 (62.8)	17 (60.7)	37 (63.8)	0.077	0.782
Diagnosis Bipolar disorder type I Bipolar disorder type II Cyclothymia	39 (45.3) 36 (41.9) 11 (12.8)	14 (50.0) 13 (46.4) 1 (3.6)	25 (43.1) 23 (39.7) 10 (17.2)	3.164	0.206
Bipolar cycle DMI MDI Irregular	20 (23.3) 17 (19.8) 49 (57.0)	7 (25.0) 0 (0.0) 21 (75.0)	13 (22.4) 17 (29.3) 28 (48.3)	10.628	0.005
Predominant polarity Depressive (Hypo)Manic Mixed	44 (51.2) 22 (25.5) 20 (23.3)	16 (57.2) 6 (21.4) 6 (21.4)	28 (48.3) 16 (27.6) 14 (24.1)	0.630	0.730
Age at onset (years)	26.47 ± 10.95	26.39 ± 9.37	26.50 ± 11.72	0.042	0.966
Duration of illness (years)	22.03 ± 13.62	26.57 ± 13.21	19.84 ± 13.38	−2.194	0.031
Psychotic symptoms	29 (33.7)	11 (39.3)	18 (31.0)	0.575	0.448
Residual symptoms	33 (38.4)	15 (53.6)	18 (31.0)	4.056	0.044
Number of hospitalizations > 5	31 (36.0)	16 (57.1)	15 (25.9)	8.015	0.005
Duration of hospitalization	16.31 ± 9.15	18.50 ± 11.91	15.26 ± 7.36	−1.552	0.124
Lifetime involuntary hospitalization	27 (31.7)	9 (32.1)	18 (31.0)	0.011	0.917
Current suicide ideation	21 (24.4)	6 (21.4)	15 (25.9)	0.201	0.654
Lifetime suicide attempts	20 (23.3)	5 (17.9)	15 (25.9)	0.754	0.686
Lifetime non suicidal self-injuries	16 (18.6)	3 (10.7)	13 (22.4)	1.707	0.191
Comorbidities, N (%)					
Medical comorbidity	45 (52.9)	18 (64.3)	27 (47.4)	2.157	0.142
Psychiatric comorbidity	38 (44.2)	12 (42.9)	26 (44.8)	0.030	0.863
Presence of one illicit substance	41 (47.7)	16 (57.1)	25 (43.1)	1.492	0.222
Nicotine	55 (64.0)	18 (64.3)	37 (63.8)	0.002	0.964
Alcohol	33 (38.4)	17 (60.7)	16 (27.6)	8.764	0.003
Cannabinoids	24 (27.9)	8 (28.6)	16 (27.6)	0.009	0.924
Psychostimulants	20 (23.3)	7 (25.0)	13 (22.4)	0.071	0.790
Pharmacological treatment, N (%)					
Polypharmacy complex	42 (48.8)	18 (64.3)	24 (41.4)	3.965	0.046
Number of medications	3.83 ± 1.30	4.18 ± 1.47	3.66 ± 1.19	−1.757	0.081
Metabolic parameters, N (%) or mean ± SD					
Physical activity (Yes)	41 (47.7)	8 (28.6)	33 (56.9)	6.073	0.014
Weight in Kg	71.90 ± 14.96	80.00 ± 16.73	67.99 ± 12.38	−3.747	<0.001
Height in m	1.68 ± 0.81	1.68 ± 0.86	1.67 ± 0.79	−0.057	0.954
Body mass index	25.56 ± 4.75	28.35 ± 4.91	24.21 ± 4.08	−4.115	<0.001
Systolic pressure (mmHg)	126.53 ± 12.00	130.11 ± 10.20	124.81 ± 12.50	−1.949	0.055
Diastolic pressure (mmHg)	75.43 ± 10.81	77.64 ± 10.01	74.36 ± 11.10	−1.325	0.189
Total cholesterol	186.86 ± 49.33	200.30 ± 59.98	179.97 ± 42.09	−1.515	0.135
High density lipoprotein cholesterol	49.68 ± 16.89	40.90 ± 14.67	54.56 ± 16.21	3.122	0.003
Low density lipoprotein cholesterol	113.32 ± 38.35	119.20 ± 42.87	110.06 ± 35.81	−0.853	0.397
Triglycerides	127.42 ± 69.50	160.82 ± 77.01	105.58 ± 55.35	−2.739	0.009

## Data Availability

The data presented in this study is available on request from the corresponding author. The data is not publicly available due to privacy/ethical restrictions.

## Supplementary Materials

The following supporting information can be downloaded at: https://www.mdpi.com/article/10.3390/jpm16010047/s1, Table S1: Multivariable logistic regression for factors associated with IR (HOMA-IR ≥ 2.5) in patients with bipolar disorder (N = 86).

## Abbreviations

The following abbreviations are used in this manuscript:

BD	Bipolar Disorder
BDNF	Brain-derived neurotrophic factor
CCI	Charlson Comorbidity Index
CI	Confident interval
CRP	C reactive protein
HDL	High density lipoprotein
HOMA	Homeostasis Model Assessment
HPA	Hypothalamic–pituitary–adrenal
IL	Interleukin
IR	Insulin resistance
LDL	Low density lipoprotein
MetS	Metabolic syndrome
OR	Odd ratio
SGA	Second-generation antipsychotics
TC	Total cholesterol
TG	Triglyceride
TNF	Tumor necrosis factor
