# Supplementary material for: Insulin Resistance in Bipolar Disorder: A Real-World Cross-Sectional Study
Source: J Pers Med. 2026 Jan 12;16(1):47. doi: 10.3390/jpm16010047 (PMC12843439; doi:10.3390/jpm16010047)
Supplement: Supplementary file 1 [file jpm-16-00047-s001.zip › jpm-3990138-supplementary.pdf]

Table S1. Multivariable logistic regression for factors associated with IR (HOMA-IR ≥ 2.5) in patients with bipolar disorder (N = 86).

	Predictor	β (SE)	S.E.	Wald	p-value	OR	95% CI
Phase 1	Body mass index (kg/m²)	.370	.135	7.576	.006	1.448	1.113–1.885
	Constant	-10.112	3.580	7.978	.005		
Phase 2	Body mass index (kg/m²)	.371	.156	5.633	.018	1.450	1.067-1.970
	Number of hospitalizations > 5 (yes vs. no)	1.857	.839	4.903	.027	6.405	1.238–33.148
	Constant	-11.024	4.286	6.614	.010		

Abbreviations: CI, confidence interval; HOMA-IR, Homeostasis Model Assessment of Insulin Resistance; IR: insulin resistance; OR, odds ratio; SE, standard error.

Candidate predictors entered in the initial model: age, sex, irregular bipolar cycle, presence of residual symptoms, number of hospitalizations > 5, daily alcohol use, presence of polypharmacotherapy, weight in kg, body mass index, physical activity, HDL cholesterol, triglycerides.

Notes: β and SE were derived from the reported OR and 95% CI (Wald approximation) because full coefficient outputs were not available in the manuscript files. With 28 events (IR cases) and 8 candidate predictors, events-per-variable (EPV) was 3.5; therefore, the model should be interpreted as exploratory/hypothesis-generating.
